# Assessment of the health of Americans: the average health-related quality of life and its inequality across individuals and groups

**DOI:** 10.1186/1478-7954-3-7

**Published:** 2005-07-13

**Authors:** Yukiko Asada

**Affiliations:** 1 Department of Community Health and Epidemiology, Faculty of Medicine, Dalhousie, University, 5790 University Avenue, Halifax, Nova Scotia, B3H 1V7, Canada

## Abstract

**Background:**

The assessment of population health has traditionally relied on the population's average health measured by mortality related indicators. Researchers have increasingly recognized the importance of including information on health inequality and health-related quality of life (HRQL) in the assessment of population health. The objective of this study is to assess the health of Americans in the 1990s by describing the average HRQL and its inequality across individuals and groups.

**Methods:**

This study uses the 1990 and 1995 National Health Interview Survey from the United States. The measure of HRQL is the Health and Activity Limitation Index (HALex). The measure of health inequality across individuals is the Gini coefficient. This study provides confidence intervals (CI) for the Gini coefficient by a bootstrap method. To describe health inequality by group, this study decomposes the overall Gini coefficient into the between-group, within-group, and overlap Gini coefficient using race (White, Black, and other) as an example. This study looks at how much contribution the overlap Gini coefficient makes to the overall Gini coefficient, in addition to the absolute mean differences between groups.

**Results:**

The average HALex was the same in 1990 (0.87, 95% CI: 0.87, 0.88) and 1995 (0.87, 95% CI: 0.86, 0.87). The Gini coefficient for the HALex distribution across individuals was greater in 1995 (0.097, 95% CI: 0.096, 0.099) than 1990 (0.092, 95% CI: 0.091, 0.094). Differences in the average HALex between all racial groups were the same in 1995 as 1990. The contribution of the overlap to the overall Gini coefficient was greater in 1995 than in 1990 by 2.4%. In both years, inequality between racial groups accounted only for 4–5% of overall inequality.

**Conclusion:**

The average HRQL of Americans was the same in 1990 and 1995, but inequality in HRQL across individuals was greater in 1995 than 1990. Inequality in HRQL by race was smaller in 1995 than 1990 because race had smaller effect on the way health was distributed in 1995 than 1990. Analysis of the average HRQL and its inequality provides information on the health of a population invisible in the traditional analysis of population health.

## Introduction

To assess the health of a population, we have traditionally relied on the average or overall level of health in a population. For example, 77.2 years of life expectancy for Americans in 2001 [1] or an infant mortality rate of 6.8 per 1,000 in the United States in 2001 [2] provide some information about the health of Americans. But the average or overall health is, by definition, one number from a population and arguably artificial. Whose level of health does the average or overall health really represent?

Researchers and policy-makers have increasingly paid attention to health inequality as an indicator of population health [3-5]. They believe that a traditional average health of a population does not provide enough information as a population health measure, and investigation of the distribution of health within a population is necessary. Thus, such policy documents as *Healthy People 2010 *[5], the national health plan for the decade in the US, and *The World Health Report 2000 *[4] clearly state two goals of improving population health: the increase in the average or overall level of health and the decrease in health inequality or disparity.

Concurrently, researchers and policy-makers have increasingly recognized the importance of health-related quality of life (HRQL) in the assessment of population health. We have traditionally measured population health with indicators of life years or mortality. These are the most robust measures of health for their objectivity and availability. Yet we value both living long and living well [6], and researchers have developed various HRQL measures to capture the value for living well [7]. *Healthy People 2010*, for example, states the importance of looking at HRQL in the assessment of how healthy Americans are [5].

Little research has incorporated both of these two interests in health inequality and HRQL into the assessment of population health [8-10]. This study assesses the health of Americans in terms of the average HRQL and its inequality, using the 1990 and 1995 National Health Interview Survey (NHIS). This study uses the Health and Activity Limitation Index (HALex) as a measure of HRQL. The use of the HALex for this present study is particularly suitable, since the HALex was developed to monitor the health of Americans during the 1990s [11]. One of the three goals of *Healthy People 2000 *is to increase the span of healthy life for Americans [12]. To assist this goal, Erickson and her colleagues created a new health measure, Years of Healthy Life (YHL) [11]. The YHL combines information on HRQL and mortality. To obtain HRQL information, Erickson and her colleagues developed the HALex based on two questions from the NHIS, activity limitation and self-perceived health. Although the HALex is one component of the YHL, researchers have used it independently as a useful measure of HRQL [13-15].

This study measures inequality in HRQL across groups as well as individuals. Researchers and policy-makers have traditionally measured health inequality across groups, for example, by income, education, occupation, race, or geographic location. Recently researchers at the World Health Organization (WHO) proposed to measure health inequality across individuals, irrespective of individuals' group affiliations, in much the same way as measuring income inequality [16]. The group and individual approaches measure different dimensions of health inequality and could yield different results [17]. The group and individual approaches can complement each other and strengthen the assessment of population health [18].

Methodologically, this study explores two recent advancements in empirical health inequality research. First, it provides confidence intervals for the degree of health inequality estimated. Although a few pioneer studies exist [4,18,19], statistical inference has yet to become a standard practice in health inequality analysis. Without statistical inference, we cannot conclude with confidence whether health inequality increased or decreased. Statisticians have developed bootstrap methods to overcome difficulties in estimating the standard error of the degree of inequality using data with a complex survey design, like the NHIS [20-22]. This study employs such methods.

Second, this study examines health inequality across groups by decomposing overall inequality into inequality within each group, inequality between groups, and inequality overlapping groups. This decomposition technique is common in analysis of income inequality and poverty [23-27]. It provides richer information than the conventional analysis of comparing the average health between groups, as recent health studies show [18,28-30]. To explore the decomposition technique, this study uses race as an example. Although this study does not intend to undertake a full investigation of health inequality by race in the US, the focus on race is compatible with the recent extension of the attention from inequalities in health care by race [31,32] to inequalities in health outcomes by race [33,34].

In this study, "health distribution" is a way in which health is spread among individuals or groups of people in a population. "Health equality" suggests the health distribution in which health is spread equally to every chosen unit of analysis. "Health inequality" means all health distributions that are otherwise. I synonymously use such terms as "inequality," "disparity," and "difference."

## Methods

### Sample and Data

Data come from the 1990 and 1995 National Health Interview Survey (NHIS) [35,36]. I select these study years because the questionnaire design of the 1990 and 1995 NHIS permits the construction of the Health and Activity Limitation Index (HALex), the health variable of this present study, exactly as proposed by its developers (see below) [11]. The NHIS uses a stratified multistage probability design, yielding a nationally representative sample of the civilian non-institutionalized US population. The method of data collection is face-to-face household interview. The interviewers obtain surrogate information for children younger than 17 years of age and persons absent at the time of the interview. The response rate is over 95%. I exclude observations missing an answer to a question necessary to construct the HALex (0.5% missing in 1990, 1.2% missing in 1995). The sample size for this study is 119,003 (1990) and 101,277 (1995). Table 1 shows the unweighted number of observations by age group (0–14, 15–24, 25–44, 45–64, 65+ years of age), sex, and race (White, Black, and other racial groups (Aleut, Eskimo or American Indian, Asian or Pacific Islander, and any other race not listed separately)) in 1990 and 1995.

**Table 1:** Description of Sample

	**1990**		**1995**	
	
	**N**	**%**	**N**	**%**
All Ages	119003	100	101277	100

Age, y				
0–14	27822	23.4	24661	24.4
15–24	16289	13.7	13510	13.3
25–44	37886	31.8	31435	31.0
45–64	22487	18.9	19834	19.6
65+	14519	12.2	11837	11.7
Men	56830	47.8	48266	47.7
Race				
White	97290	81.8	83527	82.5
Black	17886	15.0	13629	13.5
Other	3827	3.2	4121	4.1

### Measure of Health: the Health and Activity Limitation Index (HALex)

The HALex combines two types of questions collected in the NHIS, one assessing activity limitation and the other measuring self-perceived health [11]. The activity limitation questions create six categories: (1) not limited, (2) limited in other activities, (3) limited in major activity, (4) unable to perform major activity, (5) unable to perform instrumental activities of daily living, and (6) unable to perform activities of daily living. Self-perceived health is in five categories: excellent, very good, good, fair, and poor. These two items together make up a matrix of 30 combinations (Table 2).

**Table 2:** The Health and Activity Limitation Index (HALex)

		Perceived health status					
			
		Excellent	Very good	Good	Fair	Poor	Dead
			
Activity limitation	Single attribute score	1.00	0.85	0.70	0.30	0.00	
Not limited	1.00	1.00	0.92	0.84	0.63	0.47	
Limited in performing other activities	0.75	0.87	0.79	0.72	0.52	0.38	
Limited in performing major acitivities	0.65	0.81	0.74	0.67	0.48	0.34	
Unable to perform major activity	0.40	0.68	0.62	0.55	0.38	0.25	
Limited in instrumental activities of daily living (IADL)	0.20	0.57	0.51	0.45	0.29	0.17	
Limited in activities of daily living (ADL)	0.00	0.47	0.41	0.36	0.21	0.10	
Dead							0.00

Source: Erickson, Wilson, Shannon (1995)

Assignment of a score to each of these 30 combinations took three steps. Developers of the HALex first assigned a score for each of the six levels of activity limitation and the five levels of self-perceived health ("Single attribute score" in Table 2), using a mathematical technique called correspondence analysis. Correspondence analysis belongs to a family of multidimensional scaling, a technique creating a scale for a concept with multiple dimensions, for example, health consisting of mobility, sensory, cognition, emotion, and pain, or social support consisting of informational, emotional, and practical support. Correspondence analysis finds the best simultaneous representation of two domains, activity limitation and self-perceived health in the case of the HALex, by maximizing the correlation between them.

The simplest correspondence analysis applies to a two-way crosstabulation, as in the case for the HALex, activity limitation and self-perceived health. One can assign a score for each of the six levels of activity limitation by weighted least-squares where each of the six levels of activity limitation is weighted by its frequency divided by the total frequency of the six levels, and distances between each of the six levels are measured by the chi-square distance. To measure the distance between "not limited" and "limited in performing other activities" in activity limitation, for example, correspondence analysis uses the chi-square distance between these two categories by examining how people in these two categories differ with respect to the five levels of self-perceived health. Developers of the HALex conducted separate correspondence analysis for each of several different 5-year age groups and different years of the NHIS. Based on the analyses, they assigned single attribute scores for each of the two domains as listed in Table 2 that maximize the correlation between activity limitation and self-perceived health in all age groups. Please refer to Greenacre [37,38] for detail explanation of correspondence analysis.

Next, the developers of the HALex made the following assumptions. They assumed that the score for the health state with no activity limitation and excellent self-perceived health is 1.00, and the score for the health state with limited activities of daily living and poor self-perceived health is 0.10. In addition, they assumed that a health state with limited activities of daily living and excellent self-perceived health is equally bad as the health state with no activity limitation and poor self-perceived health. Based on another HRQL measure, the Health Utilities Index Mark I, they assigned the score of 0.47 for these two health states.

Finally, using the scores assigned for each level of activity limitation and self-perceived health, and the four scores based on the assumptions described above, the developers of the HALex calculated scores for the rest of the 26 health states. The formula of this calculation is based on multiattribute utility theory. Multiattribute utility theory extends the traditional expected utility theory, a theory of rational decision making under uncertainty, by adding an independence assumption. The developers of the HALex, in particular, assumed mutual utility independence, that is, health domains other than self-perceived health and activity limitation (for example, pain, emotion, or hearing) have no effect on the HALex score. For example, the HALex score for the health state with limited activities of daily living and excellent self-perceived health is 0.47 regardless of the existence of pain or emotional or hearing problems. Due to this mutual utility independence assumption, the developers of the HALex used a multiplicative function for calculating the HALex scores. Drummond et al. [39] gives detail explanation of multiattribute utility theory, and technical notes of the YHL [11] provide the further detail of the HALex construction.

Erickson has later evaluated and confirmed the construct validity of the HALex [40]. For the following health inequality analysis, I assign a HALex score to each observation in the 1990 and 1995 data.

### Measure of Health Inequality: the Gini Coefficient

A measure of health inequality summarizes a health distribution into one number. This facilitates comparison and examination by quantifying a degree of health inequality. This study uses the Gini coefficient as the measure of health inequality. The Gini coefficient has most frequently been applied to income distribution, but it is possible to apply it to health distribution as previous studies demonstrated [41,42].

**Figure 1:**
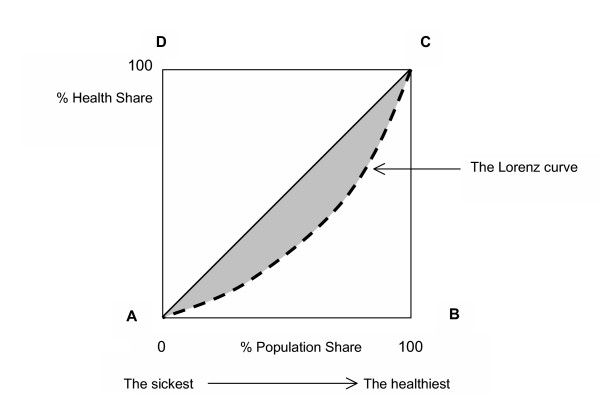
The Lorenz curve.

Figure 1 explains the Gini coefficient and the Lorenz curve (see Figure 1). Imagine that we horizontally line up individuals in a population from the sickest to the healthiest and vertically line up these individuals' health share, in the case of this present study, the cumulative percentage of the HALex. The resulting dotted curve AC is called the Lorenz curve. When the population is perfectly equal, the Lorenz curve is the diagonal line, AC. When the population is most unequal, that is, in the case of this present study, one person has a HALex score equal to or greater than 0.1 and the HALex of all others is zero (dead), the Lorenz curve follows AB and BC. The Gini coefficient is the shaded area in the graph divided by the triangle, ABC. It can take a value between zero when the Lorenz curve is diagonal, thus, perfectly equal, and one when the Lorenz curve follows AB and BC, the most unequal.

Arithmetically, the Gini coefficient (G) is expressed as:



Where the target population holds *n *people, *y*_*i *_is the HALex score of individual *i*, *y*_*j *_is the HALex score of individual *j*, and the average HALex in the population is *μ*.

#### Subgroup Decomposition of the Gini Coefficient

**Figure 2:**
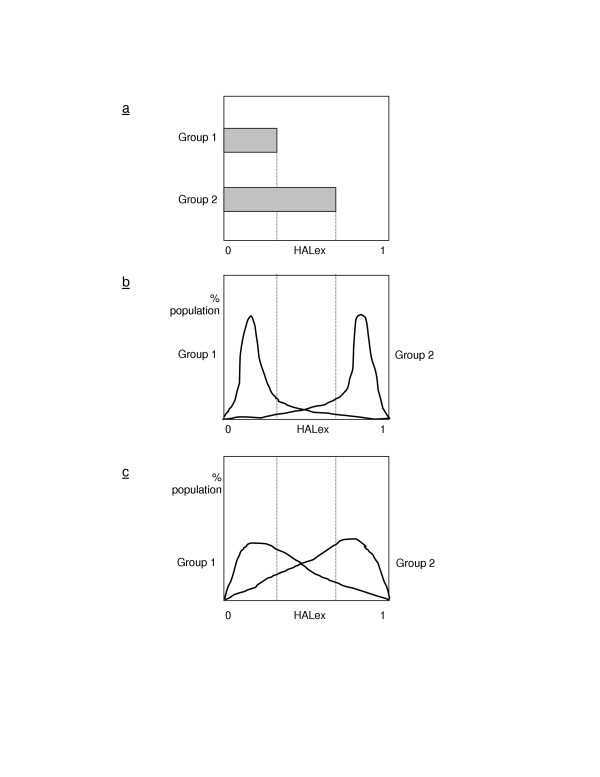
Mean difference (a), small overlap (b), and big overlap (c). Suppose we are here interested in two groups. Conventionally we compare the average health of these two groups (Figure 2a). But the same average health could come from different distributions (Figures 2b and 2c). Although Figures 2b and 2c have the same average health, the overlap between groups is greater in Figure 2c than Figure 2b. A greater overlap indicates that the group characteristic does not have much effect on the way health is distributed. These figures are not based on actual distributions and used only for illustrative purposes.

Customarily, the measurement of health inequality by group is the difference between the average health of groups (Figure 2a, see Figure 2). But the use of averages is questionable, especially when a health distribution does not follow a normal distribution. Figures 2b and 2c schematically illustrate that the same mean difference in health by group in Figure 2a could come from different distributions. The degree of overlap between the two groups in Figure 2b is smaller than that of Figure 2c. Despite the same absolute mean difference, the extent of group stratification or isolation with respect to health is greater in Figure 2b than Figure 2c. This present study adds this overlap information to the conventional absolute mean difference in analyzing health inequality by race.

Suppose we have subpopulation *k *= 1,2,.., *n*. Decomposition of the Gini coefficient (G) by subpopulation can be expressed as follows [24]:

*G *= *G*_*B *_+ ∑ *a*_*k *_*G*_*k *_+*G*_*O*_

Where *G*_*B *_is the between-group Gini coefficient, calculated under the assumption that everybody's health in subpopulation *k *is the average health of subpopulation *k*. *G*_*k *_is the Gini coefficient within subpopulation *k*. Each of this within-group Gini coefficient is weighted by population share and health share of subpopulation *k*, and its sum for all subpopulations ∑*a*_*k *_*G*_*k *_is the total within-group Gini coefficient. *G*_*O *_is a residual, which can be interpreted as the overlap Gini coefficient. When subpopulations do not overlap, *G*_*O *_equals to zero. When subpopulations are identical, that is, subpopulations perfectly overlap, *G*_*O *_also equals to zero. Unless subpopulations are perfectly identical, a greater value of *G*_*O *_suggests a higher degree of overlap of subpopulations. A higher degree of subpopulation overlap indicates that the group characteristic does not have much effect on the way health is distributed. The analysis of health inequality by race in this present study looks at how much contribution (expressed in percentage) this overlap term makes to the overall Gini coefficient, in addition to the absolute mean difference between groups.

### Analysis

This study consists of three parts: (1) analysis of the average HALex, (2) analysis of inequality in the HALex across individuals, and (3) analysis of inequality in the HALex by race. All three parts use both 1990 and 1995 data. I provide 95% confidence intervals (CI) for the average HALex using linearization (Taylor Series) methods [43,44].

Providing 95% CI for the Gini coefficient in this present study faces two difficulties. First, the Gini coefficient is a non-linear function and bounded between zero and one, which makes it difficult to use asymptotic theory. Second, the NHIS uses a complex survey design involving stratification, clustering, and multistage sampling. To overcome these two difficulties, I use a bootstrap method modified for survey data with the complex design: the two-stage with-replacement bootstrap [20-22]. Bootstrap is a simulation method only using data at hand. With a few assumptions, it can estimate the standard error for any statistic. The original bootstrap proposed by Efron and Tibshirani [45] assumes independence of observations, thus, without modification, cannot be legitimately applied to data using a complex survey design. Modification of the original bootstrap has been suggested for variance estimation of a complex survey design [46,47]. I use one of the modified versions of bootstrap, the two-stage with-replacement bootstrap, where a bootstrap sample with the sampling weight is randomly selected with replacement in two stages. McCarthy and Snowden showed that the with-replacement bootstrap yields more favourable variance estimation and CIs in stratified cluster sampling designs than the without-replacement bootstrap, another bootstrap method modified for complex surveys, where one creates an artificial population from the sample and repeatedly and randomly draws samples without replacement [21]. I repeat the simulation process 2000 times and use the percentile method to obtain 95%CI.

All analyses use weighted data. I use Stata software to conduct all analyses [48].

## Results

### The Average HALex

The assessment of the health of Americans in 1990 and 1995 differs in terms of the average HALex and life expectancy. Table 3 presents the average HALex, its 95% CI, and life expectancy [49,50] of the US population in 1990 and 1995 by sex and age group. The average HALex for both sexes (0.87 in both years), men (0.88 in 1990, 0.87 in 1995), and women (0.87 in 1990, 0.86 in 1995) of these two years were not statistically significantly different at the 5% level. Life expectancy, on the other hand, was higher in 1995 than 1990 by 0.4-year for both sexes, 0.7-year for men, and 0.1-year for women.

**Table 3:** The Average HALex and Life Expectancy in the US, 1990 and 1995

	**Both sexes (95% CI)**	**Male (95% CI)**	**Female (95% CI)**
**Life expectancy, y**			
1990	75.4	71.8	78.8
1995	75.8	72.5	78.9
**The Average HALex**			
All ages			
1990	0.87 (0.87, 0.88)	0.88 (0.88, 0.88)	0.87 (0.86, 0.87)
1995	0.87 (0.86, 0.87)	0.87 (0.87, 0.88)	0.86 (0.86, 0.86)
0–14 years			
1990	0.93 (0.93, 0.93)	0.93 (0.93, 0.93)	0.94 (0.93, 0.94)
1995	0.93 (0.93, 0.93)	0.93 (0.92, 0.93)	0.94 (0.93, 0.94)
15–24 years			
1990	0.92 (0.92, 0.92)	0.93 (0.92, 0.93)	0.91 (0.91, 0.91)
1995	0.91 (0.91, 0.91)	0.92 (0.92, 0.92)	0.91 (0.90, 0.91)
25–44 years			
1990	0.90 (0.90, 0.90)	0.90 (0.90, 0.91)	0.89 (0.89, 0.89)
1995	0.89 (0.88, 0.89)	0.9 (0.89, 0.90)	0.88 (0.88, 0.88)
45–64 years			
1990	0.82 (0.82, 0.83)	0.83 (0.82, 0.83)	0.82 (0.81, 0.82)
1995	0.81 (0.81, 0.82)	0.82 (0.82, 0.83)	0.81 (0.80, 0.81)
65+ years			
1990	0.73 (0.72, 0.74)	0.74 (0.74, 0.75)	0.72 (0.71, 0.73)
1995	0.73 (0.72, 0.73)	0.73 (0.73, 0.74)	0.72 (0.71, 0.73)

**Figure 3:**
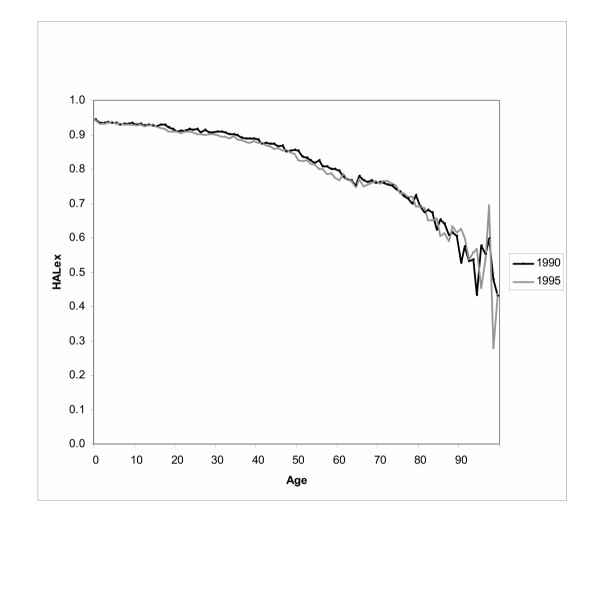
The average HALex by age in 1990 and 1995.

Figure 3 shows that the difference between the HALex in 1990 and 1995 was not consistent at every age (see Figure 3). The average HALex for both sexes was lower among 15–24 and 25–44 year olds in 1995 than 1990 (p < 0.05). Differences between the average HALex in 1990 and 1995 within other age groups were not statistically significant at the 5% level.

In both 1990 and 1995, overall women's HALex was lower than men's (0.01 difference, p < 0.05 both in 1990 and 1995). This was true in all age groups, except among 0–14 year olds both in 1990 and 1995, 45–64 year olds in 1990, and 65 year olds and older in 1995.

### Inequality in the HALex

Inequality in the HALex across individuals was greater in 1995 than 1990. Table 4 presents the Gini coefficient and its 95% CI for the US population in these years by sex and age group. The Gini coefficient was slightly greater for both sexes in 1995 than 1990 (0.005 increase for both sexes combined, p < 0.05, 0.005 increase for men, p < 0.05, and 0.004 increase for women, p < 0.05). Stratified by age group, the Gini coefficient was greater in 1995 than 1990 only for 25–44 year olds (0.007 increase for both sexes combined and both for male and female young adults, p < 0.05).

**Table 4:** The Gini Coefficient in the US in 1990 and 1995

	**Both sexes (95% CI)**	**Male (95% CI)**	**Female (95% CI)**
All ages			
1990	0.092 (0.091, 0.094)	0.087 (0.086, 0.088)	0.097 (0.096, 0.099)
1995	0.097 (0.096, 0.099)	0.092 (0.090, 0.095)	0.101 (0.100, 0.103)
0–14 years			
1990	0.048 (0.047, 0.049)	0.049 (0.048, 0.051)	0.046 (0.044, 0.048)
1995	0.049 (0.048, 0.050)	0.052 (0.050, 0.054)	0.046 (0.044, 0.048)
15–24 years			
1990	0.056 (0.054, 0.059)	0.053 (0.050, 0.056)	0.059 (0.056, 0.062)
1995	0.060 (0.058, 0.062)	0.056 (0.053, 0.059)	0.063 (0.060, 0.066)
25–44 years			
1990	0.072 (0.071, 0.074)	0.069 (0.067, 0.071)	0.075 (0.073, 0.077)
1995	0.079 (0.077, 0.081)	0.076 (0.074, 0.079)	0.082 (0.079, 0.084)
45–64 years			
1990	0.127 (0.124, 0.130)	0.126 (0.122, 0.131)	0.127 (0.124, 0.131)
1995	0.132 (0.128, 0.138)	0.130 (0.123, 0.137)	0.134 (0.130, 0.140)
65+ years			
1990	0.183 (0.178, 0.188)	0.172 (0.166, 0.178)	0.190 (0.184, 0.196)
1995	0.183 (0.174, 0.188)	0.174 (0.166, 0.183)	0.189 (0.177, 0.197)

### Inequality in the HALex by Race

Inequality in the HALex by race was smaller in 1995 than 1990 because race had smaller effect on the way health was distributed in 1995 than 1990 while the absolute mean differences between racial groups were the same between these years. Table 5 summarizes the average HALex, the Gini coefficient, and their 95% CI, and the Gini coefficient decomposed for Whites, Blacks, and other racial groups in these years. The average HALex was lower in 1995 than 1990 in all three racial groups, although only the difference among Whites was statistically significant (p < 0.05). Differences in the average HALex between all racial groups were the same in 1995 and 1990. Despite no difference in the average HALex between racial groups, the contribution of the overlap to the overall Gini coefficient was greater in 1995 than in 1990 by 2.4%.

**Table 5:** The Average HALex, the Gini Coefficient by Race in 1990 and 1995

	**1990**	**1995**
Average HALex (95% CI)		
All	0.87 (0.87, 0.88)	0.87 (0.86, 0.87)
White	0.88 (0.88, 0.88)	0.87 (0.87, 0.87)
Black	0.845 (0.84, 0.85)	0.84 (0.84, 0.85)
Other	0.89 (0.88, 0.90)	0.88 (0.87, 0.89)
Average HALex difference (* p < 0.05)		
White – Black	0.03*	0.03*
Other – Black	0.04*	0.04*
Other – White	0.01	0.010
The Gini coefficient (95% CI)		
All	0.092 (0.091, 0.094)	0.097 (0.096, 0.099)
White	0.090 (0.089, 0.092)	0.095 (0.093, 0.098)
Black	0.109 (0.104, 0.115)	0.112 (0.109, 0.116)
Other	0.077 (0.072, 0.083)	0.085 (0.080, 0.092)
Decomposition of the Gini coefficient (Contribution, %)		
Overall	0.092 (100)	0.097 (100)
Between-group	0.004 (4.72)	0.004 (4.17)
Within-group	0.066 (71.47)	0.068 (69.69)
Overlap	0.022 (23.81)	0.025 (26.15)

Health inequality between racial groups only minimally explains overall health inequality. The between-group Gini coefficient explains only 4.7% (in 1990) and 4.2% (in 1995) of the overall Gini coefficient.

## Discussion

This study showed that the average HALex of Americans in 1990 and 1995 were the same (0.87), but inequality in the HALex across individuals was slightly greater in 1995 (the Gini coefficient: 0.097) than 1990 (0.092) (p < 0.05). This study explored decomposition of the Gini coefficient as a tool to examine health inequality by group using race as an example. The decomposition analysis showed that inequality in the HALex by race was smaller in 1995 than 1990 because race had smaller effect on the way health was distributed in 1995 than 1990. Moreover, the decomposition analysis suggested that inequality in the HALex between racial groups explains only 4.7% (in 1990) and 4.2% (in 1995) of overall inequality in the HALex.

This study confirmed that one obtains different pictures of the health of a population when measuring it by life years and HRQL [4,51]. According to this study, the average HALex of Americans was the same in 1990 and 1995, although their life expectancy was higher in 1995 than 1990. This study only compared the average HALex of two years, 1990 and 1995, thus, it does not provide information of a trend of the HALex. Routine collection of the average HALex of Americans along with measures of mortality or life year will enable a richer assessment of the health of Americans. Moreover, reporting of an age-standardized HALex can show changes in the HALex independent from the age structure of the populations.

For the wider use of the HALex in the assessment of the health of Americans, future work should acknowledge that the HALex is derived from self-reported activity limitation and self-perceived health questions. Should we assess population health based on a self-reported measure of health such as the HALex or on an "objective" measure of health such as medical diagnosis? Observation of the differences in the HALex between men and women in this study suggests the importance of this question. This study showed that in both 1990 and 1995, women's HALex was lower than men's in all age groups, except among children, 0–14 years old. All of these differences in the HALex by sex, except among 45–64 year olds in 1990 and 65 year olds and older in 1995, are statistically significant at the 5% level. In contrast, life expectancy was 7 years higher for women than men in 1990, and 6.4 years higher for women than men in 1995. In addition, the WHO reports that healthy life expectancy, which combines life expectancy and HRQL, was 4.1 years higher for women (71.3 years) than men (67.2 years) in 2002 [52]. Is women's health status "objectively" lower than men's, or do women perceive their health status lower than men's? What if we discovered that women perceive the same, "objective" health conditions lower than men – should we consider low perception as a health problem? The issue of perception is not only limited to sex but also applies to socioeconomic status, racial groups, or geographic location. The future work needs to investigate how much of the difference in the HALex is due to the difference in perception and identify appropriateness of using the HALex or any other self-reported measure of health in the assessment of population health.

This study was the first to describe inequality in the HRQL among a nationally representative sample of Americans. It showed that inequality in the HALex across individuals in the US was greater in 1995 than 1990. Although this descriptive study cannot explain reasons for this difference, one possible speculation for this finding is that the increasing income inequality due to uneven distribution of the economic growth [53,54] might have an effect on health inequality.

This study used the Gini coefficient as the measurement of health inequality. Following the previous studies that applied the Gini coefficient to health distribution [41,42], this study reported three-decimal Gini coefficients. However, an appropriate level of precision of the Gini coefficient used in health distribution, especially distribution of the HALex, is unknown. Future work needs to investigate this point.

Age group analysis uncovered the worrisome health of young Americans, especially 25–44 year olds. The average HALex was lower and its inequality was greater in 1995 than 1990 in this age group. A possible etiology for this finding is the spread of HIV/AIDS. In 1995, HIV/AIDS was the leading cause of death among the young [55]. Another factor might be the general trend that the onset of chronic diseases has shifted to younger ages. Even without these epidemiologic trends, one might call the age group of 25–44 year olds the "forgotten" age group. Health policy tends to focus on infants, adolescents, and the elderly. Young adulthood is often considered as the most resilient stage of life in terms of human biology. It is, however, this period of life in which the proportion of the uninsured is the second highest (after 18–24 year olds) [56], and young families struggle to establish themselves.

This study explored the subgroup decomposition technique as a tool for analysis of health inequality by group. A striking finding from the decomposition analysis was that only 4–5% reduction of overall inequality would be possible even if differences in the mean HALex between all racial groups disappeared. The importance of race for social justice considerations does not depend on the magnitude of the between-group inequality. Nonetheless, such information can be useful in examining policy implications of health inequalities by different group characteristics. In other words, the decomposition analysis helps us investigate health inequality from a broader perspective: given that differences in the HALex between racial groups only explains 4–5% of overall inequality in the HALex, what factors are accountable for the rest of 95–96% of overall inequality?

Application of the subpopulation decomposition technique to health inequality analysis is admittedly still in its infancy. This study used the subgroup decomposition technique only for one group characteristic (race) at a time. The literature on income inequality and poverty and pioneering health studies point to three possible extensions of this unidimensional subgroup decomposition. First, adjusting for a number of groups in subgroup characteristics (for example, three groups for race, and five groups for income), one can compare results of the subgroup decomposition applied to different subgroups [25,26]. This means that one can identify how much of the overall health inequality comes from, for example, health inequalities by gender, income, education, and geographic location. Second, overall health inequality can be decomposed not only by subgroup but by component [30]. An overall health state assessed by the HALex consists of two components, self-perceived health and activity limitation. By decomposing overall health inequality by component, one can identify inequality in which of these components contribute more to inequality in overall health. Finally, multidimensional decomposition is possible. One can decompose overall health inequality either jointly by subgroup and component [57] or by multiple subgroups at once (for example, race and income) [58]. The multidimensional decomposition has proven to be of great policy value in income inequality and poverty fields. With these developments, the decomposition technique is promising not only for summarizing diverse health inequality information but also for identifying determinants of health inequalities.

Although this study did not aim to investigate fully health inequality by race, the contrast between the greater health inequality across individuals and the smaller health inequality by race in 1995 than 1990 is worth emphasizing. One must receive this welcome finding with a caution. Differences in the average HALex between the three racial groups were the same in 1990 and 1995. Moreover, the greater overlap of the HALex distribution between racial groups came with the lower average HALex of all racial groups in 1995 than 1990. These findings of the HALex contrast with life years. Life expectancy both for Blacks and Whites were longer in 1995 than 1990 (Blacks: 69.1 in 1990, 76.1 in 1995, Whites: 69.6 in 1990 and 76.5 in 1995). The difference in life expectancy between Blacks and Whites was slightly smaller (0.1 year) in 1995 than 1990 because the difference in life expectancy between 1990 and 1995 was marginally greater for Blacks than Whites [49,50]. A follow-up of *Healthy People 2000 *found that all racial or ethnic groups, except American Indian or Alaska Native, reduced all-cause morality rates between 1990 and 1995 [33,34]. The categorization of racial groups is a major limitation of this study. The "other" group limits further meaningful comparisons of this study to other studies. Given that this study showed the average HALex was highest and its inequality was lowest in the "other" racial group both in 1990 and 1995, the further classification of this group would provide useful information on the health of different racial groups in the US.

## Conclusion

*Healthy People 2010 *[5] aims to improve the average or overall health of Americans and to reduce health inequality among them. According to these two goals of population health, the assessment of population health is incomplete without analyzing health inequality. This present study is one example of health inequality analyses for the assessment of the health of Americans. Just as national health statistics routinely report the average or overall health of a population, health inequality needs to be routinely reported as an indicator of the health of Americans. Future work must determine which health inequalities can best serve as national health statistics.

*Healthy People 2010 *[5] also emphasizes the importance of paying attention to HRQL in the assessment of population health. As this study showed, inclusion of HRQL in the assessment of the health of Americans enriches our knowledge of population health and stimulate health policy debate.

Although the focus of the present study is primarily American, these key messages are internationally generalizable. This study should be of interest for health researchers and policy-makers in the US and elsewhere who wish to advance the assessment of population health.

## List of Abbreviations

CI (confidence intervals)

HALex (Health and Activity Limitation Index)

HIV/AIDS (Human Immunodeficiency Virus / Acquired Immune Deficiency Syndrome)

HRQL (health-related quality of life)

NHIS (National Health Interview Survey)

US (United States of America)

WHO (World Health Organization)

## Competing Interests

The author(s) declare that they have no competing interests.

## Authors' Contributions

YA was solely responsible for this work.
